# Adaptive Models for Gene Networks

**DOI:** 10.1371/journal.pone.0031657

**Published:** 2012-02-16

**Authors:** Yong-Jun Shin, Ali H. Sayed, Xiling Shen

**Affiliations:** 1 Electrical and Computer Engineering, Cornell University, Ithaca, New York, United States of America; 2 Electrical Engineering, University of California Los Angeles, Los Angeles, California, United States of America; Center for Genomic Regulation, Spain

## Abstract

Biological systems are often treated as time-invariant by computational models that use fixed parameter values. In this study, we demonstrate that the behavior of the p53-MDM2 gene network in individual cells can be tracked using adaptive filtering algorithms and the resulting time-variant models can approximate experimental measurements more accurately than time-invariant models. Adaptive models with time-variant parameters can help reduce modeling complexity and can more realistically represent biological systems.

## Introduction

In science and engineering, computational models are needed to describe the relationship between input and output data of a system as well as to estimate future outputs based on inputs. One common approach for constructing models from measured input/output data is system identification (SI), which uses computational techniques to build models of dynamical systems using the data [1]. It is usually not feasible to build a white-box SI model, in which all necessary information about the system is available. A more practical approach is to construct a grey-box SI model, which depends on some prior knowledge about the system, or a black-box SI model, which does not require any prior knowledge about the system. Parameters of a grey-box model usually describe specific physical processes, e.g., the rate constant of a reaction, whereas parameters of a black-box model may not [1].

Gene regulatory networks are dynamical systems. Biologists regularly attempt to infer gene regulatory networks and build mathematical models based on measured signaling (protein, messenger RNA, microRNA, etc.) levels. Recent technological advancement has made it possible to perform time-lapse microscopy to track dynamical signaling states in individual cells using fluorescent reporters (reviewed in [2]). SI is thus well suited for deducing gene network models based on such measurements.

**Figure 1:**
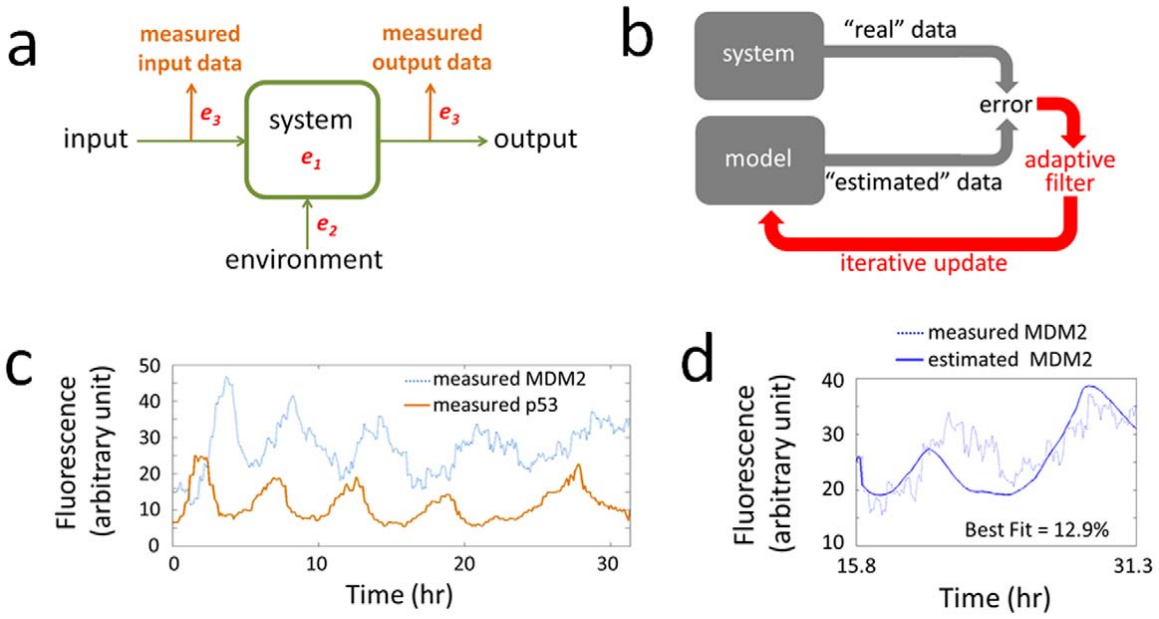
System identification of the p53-MDM2 gene network. (**a**) Models describe relationships between measured input and output data. They are subject to three types of uncertainty: system uncertainty (*e_1_*), environmental uncertainty (*e_2_*), and measurement uncertainty (*e_3_*). (**b**) An adaptive filter iteratively adjusts the model parameters based on the error between the measured and estimated data. (**c**) p53 and MDM2 levels oscillate after radiation-induced DNA damage. (**d**) The best time-invariant ARX model (*n_a_* = 1, *n_b_* = 3, *n_k_* = 2) only has a Best Fit score of 12.9%.

However, models of gene regulatory networks derived by SI have to cope with various sources of uncertainty **(** **Fig. 1a** **)**. First, knowledge of gene networks, especially their stochastic processes [3], [4], is usually incomplete, which limits the accuracy of the assumed model (*e*_1_). Second, the behavior of the network is influenced by environmental factors (*e*_2_), which are often difficult to model. Third, the observed data are subject to measurement errors (*e*_3_). All these sources of uncertainty contribute to the perceived stochasticity of gene networks preventing the model estimates from better matching the data.

To achieve a better “fit” between models and measurements, researchers often resort to increasing the order or complexity of their models [5], [6] while assuming constant model parameters [7]–[9]. However, since many of the processes underlying the uncertainties of gene networks are likely to be inherently time-variant, we hypothesize that time-variant models can potentially match and estimate experimental measurements better than time-invariant models. Furthermore, tracking the change of parameter values over time may help quantitatively approximate how time-variant gene networks behave.

In this study, we demonstrate that adaptive filtering (in engineering, the term *filter* is used to refer to a system that processes or “filters” input signals to generate output signals) techniques can be applied to creating time-variant models for gene networks [10]. Widely used in engineering disciplines such as communications, signal processing, and control, an adaptive filter iteratively and continuously adjusts the model parameters based on the error between the measured and estimated data (Fig. 1B). Using recently available time-series data for the p53-MDM2 network as an example [5], we demonstrate that adaptive filters can be used to “track” the changing parameters of gene network models and to enhance model estimation. The tumor suppressor p53 is one of the most studied proteins in cancer research [11], [12]. In cellular stress conditions such as radiation-induced DNA damage, p53 levels are reported to oscillate in a sustained manner (Fig. 1C) [13]. p53 and MDM2 form a negative feedback loop – p53 transcriptionally activates MDM2, while MDM2 degrades p53 via ubiquitination [14]. The levels of p53 and MDM2 in individual MCF7 cells have been tracked by time-lapse microscopy using the p53-CFP and MDM2-YFP fluorescent reporters [5].

## Results and Discussion

Before a model is constructed from data using SI, three choices should be made: the model structure, model order, and parameter estimation method by which a candidate model structure/order combination is assessed [1]. As illustrated later, the choice of parameter estimation method determines whether the model is adaptive or not. We use an autoregressive with exogenous input (ARX) model structure for the p53-MDM2 network (see Methods). Widely used for SI in engineering, ARX is often capable of accurately approximating and describing underlying system dynamics in real-world applications [1]. The ARX model structure is represented by a combination of three parameters: *n_a_*, *n_b_*, and *n_k_*. The model order, which reflects the model complexity, is taken to be the total number of the parameters used (the sum of *n_a_* and *n_b_*). Note that the ARX models are “discrete-time” models commonly used in engineering (signal processing) and computational physics [15], [16]. The parameter values of discrete-time difference equation models such as ARX are determined by, but do not map one-on-one to, the rate constants of physical reactions. This is different from continuous-time differential equation models, wherein each parameter directly represents the rate constant of a physical reaction. For instance, let us assume we have two genes (*u* and *y*) whose protein levels are measured every 10 minutes using time-lapse microscopy. We assume no prior knowledge about the relationship between *u* and *y*. For this case, one possible 1^st^ order discrete-time model can be:
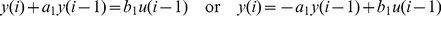
(1)Eq. 1 indicates that *y* measured at time *i* can be expressed as a linear combination of y and *u* measured at time *i-1* (10 minutes ago). As shown in Appendix S1 (Note 1), the parameters *a_1_* and *b_1_* are determined by a combination of rate constants within the 10 minute time window and each parameter does not directly represent one specific reaction. The rate constants are also related to the modes of the characteristic polynomial whose coefficients are formed from the parameters of the discrete-time model [15].

We first assume the ARX model is time-invariant, so the model has constant parameters. We proceed to find the model order that gives best estimates. For each model order, the best parameter values that fit the measured data are identified using the Least Squares estimation method (see Methods). After trying 1,000 *n_a_*, *n_b_*, and *n_k_* combinations, which includes a grey-box model (*n_a_* = 2, *n_b_* = 1, *n_k_* = 2, see Appendix S1 (Note 1) for its derivation starting from the Geva-Zatorsky's linear model [6]) that reflects prior knowledge of the negative feedback loop, it was found that the model order with the best performance is 4 (*n_a_* = 1, *n_b_* = 3, *n_k_* = 2) (Appendix S1 (Note 2)). However, Figure 1D shows that even this best model only has a score of 12.9% according to the Best Fit measure with 100% corresponding to a perfect fit and 0% corresponding to a simple average (see Methods). The Best Fit score is not improved when we applied to the same data other SI model structures such as ARMAX, output-error, and state-space (Appendix S1 (Note 3)). These results indicate that it is challenging for the time-invariant ARX model to find parameters that fit the measured data well. It is worth noting that such poor fit between models and measurements are common for gene network models.

The poor model estimates are probably caused by many factors. The p53-MDM2 dynamics are known to be influenced by other genes and proteins [11], [12]. For example, Colaluca *et al.* reported that NUMB enters into a tricomplex with p53 and MDM2, thereby preventing p53 ubiquitination [17]. Another example is the kinase ATM, which can affect the p53-MDM2 dynamics by activating p53 [6]. Hence fluctuations of protein levels such as NUMB and ATM can translate into p53 and MDM2 fluctuations, which, together with many other factors, contribute to the system uncertainty (*e_1_*). Furthermore, the dynamics are also influenced by environmental uncertainty (*e_2_*) (e.g., temperature variations and cell-cell interactions) and by measurement uncertainty (*e_3_*). These uncertainties are likely time-variant, causing the time-invariant ARX model to provide poor estimates.

Can a time-variant p53-MDM2 model improve the model performance? If so, it will indicate that the measured dynamics of the p53-MDM2 negative feedback in individual cells has a time-variant component. To test this hypothesis, we implement and compare three adaptive filtering algorithms, NLMS (Normalized Least Mean Squares), RLS (Recursive Least Squares), and Kalman filter (see Methods), which allow the model to track the changing parameters over time. NLMS is a variation of LMS (Least Mean Squares), a popular adaptive filter due to its simplicity and robustness [10]. The LMS iteration step-size 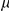 is a tradeoff among the rate of convergence, stability, and steady-state performance, and we use NLMS, which uses a self-adjustable step-size, to improve performance. The second algorithm, RLS, is computationally more intensive and usually has a faster convergence rate than NLMS. Through a “forgetting factor” 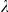, RLS can assign larger weights to recent data and smaller weights to data in the remote past, thereby enabling the algorithm to track changing systems [10]. The third is the Kalman filter, which is widely used in real engineering applications such as GPS (Global Positioning System) and the most complex algorithm among the three options studied in this work. The underlying state-space model for Kalman filtering can assume different characteristics for the biological noise (*e_2_*) and the measurement error (*e_3_*). Thus, any knowledge about the noise spectrum can be utilized to improve model performance. The three adaptive algorithms can be evaluated by readers using the program and data provided in the supporting information files (Software S1 and Data S1 (p53) and S2 (MDM2)). Instructions for using the program can be found at Appendix S1 (Note 4). See also Video S1.

**Figure 2:**
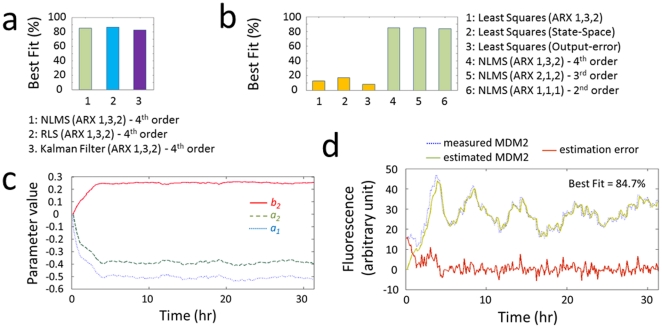
A time-variant model using adaptive filtering. (**a**) The three types of adaptive filter implementations (NLMS, RLS, and Kalman filter) achieve similar Best Fit scores (near 80%) with the 4^th^ order ARX model (*n_a_* = 1, *n_b_* = 3, *n_k_* = 2). (**b**) Adaptive filtering-based time-variant models (4, 5, and 6) outperform time-invariant models (1, 2, and 3). The performance of the adaptive filter is insensitive to the order of the model in these simulations; with NLMS, a 3^rd^ order grey-box ARX model (*n_a_* = 2, *n_b_* = 1, *n_k_* = 2) and a 2^nd^ order ARX model (*n_a_* = 1, *n_b_* = 1, *n_k_* = 1) performing as well as the 4^th^ order ARX model (*n_a_* = 1, *n_b_* = 3, *n_k_* = 2). The ARX *n_a_*, *n_b_*, and *n_k_* values are enclosed by parentheses in the figure. (**c**) Parameter tracking by the NLMS filter for the 3^rd^ order ARX model (*n_a_* = 2, *n_b_* = 1, *n_k_* = 2). Each color line represents the changing values of a single parameter. (**d**) The NLMS algorithm enables the model to closely match measurements, increasing the Best Fit score to 84.7%. The estimation errors are reduced after an initial brief “learning” period for the adaptive filter.

Using the previous 4^th^ order ARX model (*n_a_* = 1, *n_b_* = 3, *n_k_* = 2), all three adaptive filter algorithms improve the Best Fit score to around 80% (Fig. 2A) with the NLMS solution being the least computationally intensive, compared to the 12.9% achieved by the time-invariant model. Using NLMS, to find out if the model order significantly affects the performance, we tested a 3^rd^ order grey-box ARX model (*n_a_* = 2, *n_b_* = 1, *n_k_* = 2) described earlier and a simple 2^nd^ order ARX model (*n_a_* = 1, *n_b_* = 1, *n_k_* = 1). Figure 2B illustrates that adaptive filtering (NLMS)-based time-variant models (4, 5, and 6) significantly outperform time-invariant models (1, 2, and 3). It is also seen that NLMS allows the low-order (3^rd^ and 2^nd^) adaptive models (time-varying models using adaptive filtering) to achieve comparable performance to the high-order (4^th^) model. These observations suggest that the measured dynamics of the p53-MDM2 gene network has a time-variant component (*e_1_*, *e_2_*, and/or *e_3_*), which enables lower-order, time-variant models to outperform higher-order, time-invariant models. More broadly, our results suggest that the common practice of increasing model complexity without taking into account the time-variant uncertainties may not necessarily yield better estimates for gene networks.

Tracking the parameters over time provides an intuitive way for evaluating the time-variant component of the measured p53-MDM2 dynamics. Figure 2C and 2D show the results of using the NLMS algorithm for tracking the 3^rd^-order ARX model (*n_a_* = 2, *n_b_* = 1, *n_k_* = 2) parameters (see also Appendix S1 (Note 5)). In Figure 2C, it is seen that the parameter values are continuously updated to reduce the estimation error at each iteration. Each color line represents the changing values of a single parameter. The resulting fit between measurements and estimates is observed in Figure 2D - the adaptive filter iteratively estimates the MDM2 level and the corresponding Best Fit score is 84.7%. Note that there is a period of relatively large estimation errors in the initial transient phase while the filter is learning.

In this work, we demonstrate that time-variant models using adaptive filters can provide more accurate estimates of single cell measurements than time-invariant models. Taking time variation into consideration allows lower-order, simpler models to outperform higher-order, time-invariant models. SI with adaptive filters can provide a useful modeling methodology thanks to the increasing number of time-series and single cell measurements that are becoming available these days. The exact mechanisms of these systems are often not completely understood, making grey- and black-box SI models a convenient tool for estimating system behaviors. Although we introduced adaptive filtering as an estimation technique for better fitting a model to data, the same approach may be used to elucidate the adaptive behavior of biological systems. In that respect, tools from adaptive networks [18]–[20] are potentially appropriate for modeling the adaptive nature of large-scale interacting biological systems, including gene networks. Another possible extension of our work is to use adaptive filters and various forms of control mechanisms, such as linear quadratic and robust control methods, for identifying and controlling the stochastic dynamics of gene networks in real time. This approach will require designing and building synthetic gene circuit components that can function as sensors and controllers. Recent advances in fields such as systems and synthetic biology enable such applications that use *in silico* controls to regulate *in vivo* gene circuits [21].

## Methods

### Image extraction and fluorescence quantification

285 Image frames were extracted from the video file [5] and the fluorescence quantification of p53 and MDM2 was carried out using the National Instruments Vision Assistant 2010. We manually marked the location of each cell nucleus in each frame and 285 data points were obtained for each protein.

### ARX model structure

For a single-input/single-output system, the ARX model structure is represented as [1]:
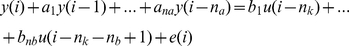
where 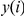 represents the output at time 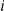, 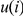 represents the input at time 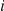, 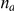 and 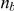 designate the number of past output and input samples that enter into the model, 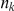 is the delay before the input affects the system output, and 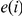 represents the uncertainty at time 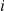.

### System identification and the Best Fit score

For SI we used the MATLAB System Identification Toolbox (Mathworks, USA) and the LabVIEW System Identification Toolkit (National Instruments, USA). For Least Squares-based time-invariant parameter estimation, the input and output data were divided into two sets of data, estimation and validation sets. Estimation data (from image frames 1 to 142) is the data set used to fit a model to the data, while validation data (from image frames 143 to 285) is the data set used for model validation purposes. For the adaptive filter implementations, the input and output data were not divided into estimation and validation sets because this division is not necessary; instead, the filters were iteratively and continuously applied to the data set.

The performance was measured using the Best Fit score and the equation for computing the score is:
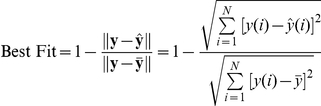
where 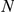 is the number of samples used (data), 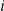 is the sample index, 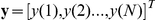, 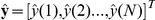, and 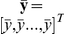 (*N* entries). 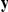 is the measured output (MDM2) vector and 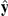 is the estimated output vector. 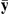 is a vector with the repeated mean 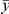 of the data 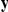. A score of 100% corresponds to a perfect fit, and a score of 0% indicates that the fit is no better than guessing the output to be the mean value 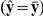. For the adaptive filtering algorithms, the Best Fit score was computed using the last 200 (out of 285) data points to exclude the initial transient effects.

In the equation-error approach, the data vector 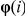 consists of 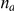 elements of the output (MDM2) data vector 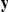 and 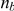 elements of the input (p53) data vector 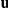 as shown below.
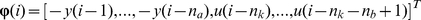



### Least Squares method

The parametric vector to be estimated is denoted by 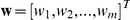 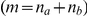, and its entries refer to the parameters 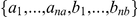 of the ARX model. The estimated output 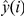 and the error 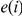 are computed using the following equations.
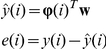
The least-squares criterion is expressed as:
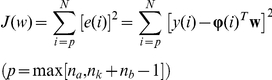
The parameter vector 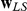 that minimizes 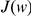 is given by:
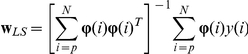



### Adaptive filtering algorithms

The parametric vector to be estimated is denoted by 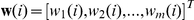 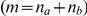, and its entries refer to the parameters 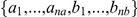 of the ARX model at each iteration. Adaptive algorithms for estimating ARX models fall into the class of adaptive IIR filters [22]. In this work, we illustrate the modeling capabilities of adaptive methods by focusing on the equation-error approach; other approaches are also possible including conditions to examine the stability of the resulting models. The estimated output 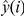 and the error 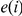 are computed using the following equations.
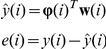



#### 1. Normalized Least Mean Squares (NLMS)

The parametric vector 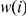 is updated according to the following equation.
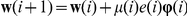
where 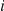 is the iteration index and 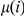 is the iteration step size at time 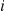.

The self-adjustable step size 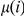 is chosen as:
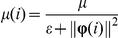
where 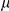 is the fixed iteration step size (0.1 was used for the simulated experiments) and 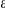 in the denominator is a very small positive constant 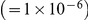 that avoids division by zero or by a small number when 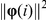 is zero or approaches zero. The correction term 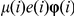 that is added to 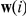 in the recursion is normalized with respect to the squared-norm of 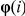. As a result, the algorithm is less affected by large fluctuations in the data. Since NLMS is obtained as a stochastic-gradient approximation to Newton's Method, NLMS exhibits a faster convergence behavior than LMS [10].

#### 2. Recursive Least Squares (RLS)

The estimated parametric vector 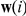 is updated according to the following equation [10].
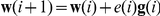
The gain vector 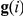 is defined by the following equation.
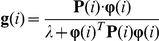
A typical range of values for the forgetting factor is 0.98<
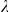
<1 and we used 0.98 for the simulated experiments. 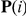 is an *m* by *m* matrix updated using the following equation.
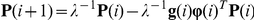
The initial condition for 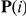 was chosen as 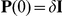, where 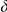 is a large number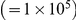 and 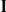 is an identity matrix (m by m).

#### 3. Kalman Filter

Similar to RLS, the estimated parametric vector 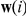 is updated according to the following equation [10].
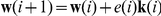
The gain vector 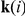 is defined by the following equation:
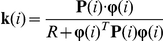
where 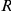 is the measurement noise variance, which is related to the observation uncertainty (measurement error) *e_3_*. The 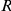 value assumed in the simulations was 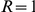. 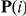 is an *m* by *m* matrix updated using the following Riccati recursion:
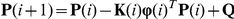
where 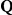 is the covariance matrix (*m* by *m*) of the process noise, which is related to the environmental uncertainty (biological noise) *e_2_*. We select 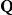 in the form:
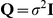
where the standard deviation is chosen as 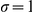 and 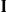 is an identity matrix (m by m). In the simulations, the initial condition for the Riccati recursion was chosen as 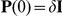, where 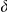 is a large number 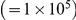.

## Supporting Information

Appendix S1 **Supplementary Notes.** Note 1: Derivation of the Grey-Box Model (*n_a_* = 2, *n_b_* = 1, *n_k_* = 2). Note 2: Finding the Best Fit ARX Model Order Using the Least Squares Estimation Method. Note 3: Comparing the Performance of Different Model Structures. Note 4: Instructions for Using AFGN.exe. Note 5: Steps for reproducing Figure 2C and 2D.
(PDF)

Software S1 **AFGN.exe.** A LabVIEW-based GUI for evaluating adaptive algorithms introduced in the main text.
(EXE)

Data S1 **p53_data.txt.** p53 fluorescence measurement data file.
(TXT)

Data S2 **mdm2_data.txt.** MDM2 fluorescence measurement data file.
(TXT)

Video S1 **A video demonstration of running AFGN.exe.**
(AVI)
