# Supplementary material for: Adaptive Models for Gene Networks
Source: PLoS One. 2012 Feb 16;7(2):e31657. doi: 10.1371/journal.pone.0031657 (PMC3280989; doi:10.1371/journal.pone.0031657)
Supplement: Appendix S1 — Supplementary Notes. Note 1: Derivation of the Grey-Box Model (na = 2, nb = 1, nk = 2). Note 2: Finding the Best Fit ARX Model Order Using the Least Squares Estimation Method. Note 3: Comparing the Performance of Different Model Structures. Note 4: Instructions for Using AFGN.exe. Note 5: Steps for reproducing Figure 2C and 2D. (PDF) [file pone.0031657.s001.pdf]

Supplementary Note 1: Derivation of the Grey-Box Model (n_a = 2, n_b = 1, n_k = 2).

An ordinary differential equation (ODE) for simple gene regulation (gene *u* activates gene *y*) can be expressed as [1]:

$$\frac{dy(t)}{dt} = \frac{F_{max}[u(t)]^n}{K^n + [u(t)]^n} - p_y y(t) \qquad \text{(Eq. 1)}$$

where $u(t)$ and $y(t)$ stand for the concentrations of protein *u* and *y* as functions of time t. F_{max} is the maximal level of the *y* protein production (in units of concentration per unit time) that is reached when $u(t) >> K$. K is the concentration of $u(t)$ at which half-maximal production of *y* protein is reached and *n* is the Hill coefficient. p_y is a degradation/dilution parameter that affects the rate at which *y* decreases. Eq. 1 can be transformed into a linearized form as [1]:

$$\frac{dy(t)}{dt} = p_{uy}u(t) - p_y y(t) \qquad \text{(Eq. 2)}$$

where p_{uy} is a parameter that determines the effect of the *u* protein on the production of the *y* protein. Denoting p53 as $u(t)$ and MDM2 as $y(t)$, the dynamics of the p53-MDM2 negative feedback loop can be expressed as [2]:

$$\frac{du(t)}{dt} = -p_{yu}y(t) - p_u u(t) \qquad \text{(Eq. 3)}$$

$$\frac{dy(t)}{dt} = p_{uy}u(t) - p_y y(t) \qquad \text{(Eq. 4)}$$

Eq. 3 and Eq. 4 can be discretized using Euler's method (*h*: discrete-time unit):

$$u(i) = (1 - p_u h)u(i-1) - p_{yu} h y(i-1) \qquad \text{(Eq. 5)}$$

$$y(i) = p_{uy} h u(i-1) + (1 - p_y h)y(i-1) \qquad \text{(Eq. 6)}$$

where i is the iteration index. By substituting Eq. 5 into Eq. 6, we get:

$$y(i) = (1 - p_y h)y(i-1) - p_{uy}p_{yu}h^2 y(i-2) + p_{uy}h(1 - p_u h)u(i-2) \qquad \text{(Eq. 7)}$$

If we further incorporate an error term $e(i)$ into Eq. 7 to model approximation errors, then we find that the discrete-time linear difference equation derived from the Geva-Zoatorsky's continuous-time linear differential equation [2] is a 3rd-order ARX model as shown below.

$$y(i) = -a_1 y(i-1) - a_2 y(i-2) + b_2 u(i-2) + e(i):\ (n_a = 2, n_b = 1, n_k = 2)$$

Supplementary Note 2: Finding the Best Fit ARX Model Order Using the Least Squares Estimation Method

Using the MATLAB System Identification Toolbox (Mathworks, USA), we tested 1,000 combinations of n_a, n_b, and n_k values that change from 1 to 10 (10 x 10 x 10 = 1,000). The model order that corresponds to the best model performance is selected based on unexplained output variance (the vertical axis in **Fig. S1**), the ratio between the prediction error variance and the output variance in percent [3]. Unexplained output variance is the portion of the output not explained by the model. The relationship between unexplained output variance and the Best Fit score can be shown as:

$$\text{unexplained output variance} = \frac{\|\mathbf{y}-\hat{\mathbf{y}}\|^2}{\|\mathbf{y}-\bar{\mathbf{y}}\|^2}$$

$$\text{Best Fit} = 1 - \frac{\|\mathbf{y}-\hat{\mathbf{y}}\|}{\|\mathbf{y}-\bar{\mathbf{y}}\|} = 1 - \sqrt{\text{unexplained output variance}}$$

where $\mathbf{y}$ is the measured output (MDM2) vector, $\hat{\mathbf{y}}$ is the estimated model output vector, and $\bar{\mathbf{y}}$ is the vector with all entries equal to $\bar{y}$, the mean of the data vector $\mathbf{y}$. The relationship illustrates that as the unexplained output variance increases the Best Fit score decreases and vice versa. For our case, the model order with the least unexplained output variance turned out to be 4 (n_a = 1, n_b = 3) (**Fig. S1**). In the figure, it is also seen that models with low orders (between 2 and 4) perform similarly, suggesting that the order may not play a critical role in that range. On the other hand, performance degrades when the order increases, indicating that unnecessarily complex models can degrade performance. Since a good model is one that is simple and performs well enough, we may choose small model orders such as 2 or 3 in real applications.

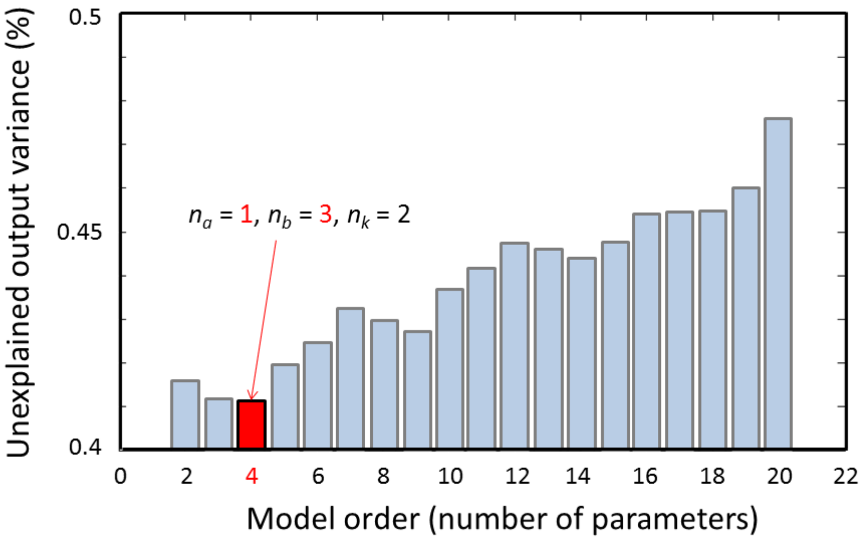

Figure S1. Model order vs. unexplained output variance (estimation error measure).

The equation and parameter values of the 4th order model (n_a = 1, n_b = 3, n_k = 2) are shown below.

$$y(i)+a_1 y(i-1)=b_2 u(i-2)+b_3 u(i-3)+b_4 u(i-4)+e(i)$$

a_1 = -0.9491

b_2 = -0.03745, b_3 = 0.05409, b_4 = 0.1097

The equation and parameter values of the 3rd order model (n_a = 2, n_b = 1, n_k = 2) described in **Supplementary Note 1** are shown below.

$$y(i)+a_1 y(i-1)+a_2 y(i-2)=b_2 u(i-2)+e(i)$$

a_1 = -1.038, a_2 = 0.07941

b_2 = 0.1053

Supplementary Note 3: Comparing the Performance of Different Model Structures

Figure S2 illustrates that performance is not observed to improve with other commonly used model structures such as ARMAX, Box-Jenkins, output-error, and state-space (the Best Fit scores are shown in the parentheses) [3]. This suggests that choosing different model structures is not sufficient to resolve the issue of increasing the Best Fit score.

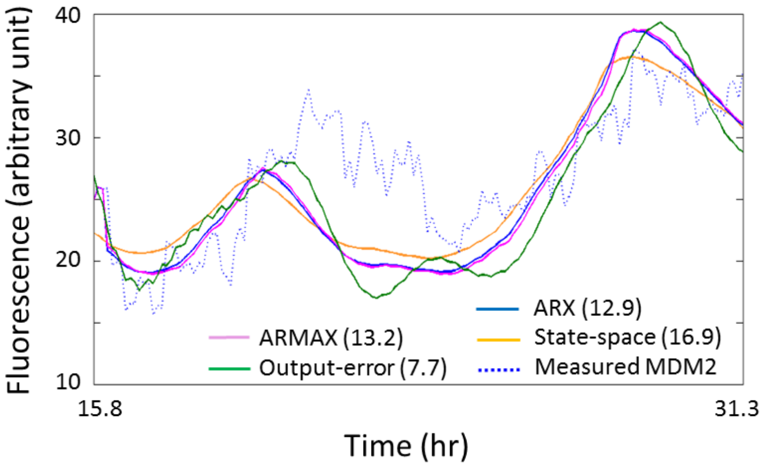

Figure S2. Performance of different model structures (the percent Best Fit scores are shown in the parentheses).

Supplementary Note 4: Instructions for Using AFGN.exe

As a supplementary material, we provide a LabVIEW-based Windows application that can be used to run the simulated experiments described in the main text (**Fig. S3**).

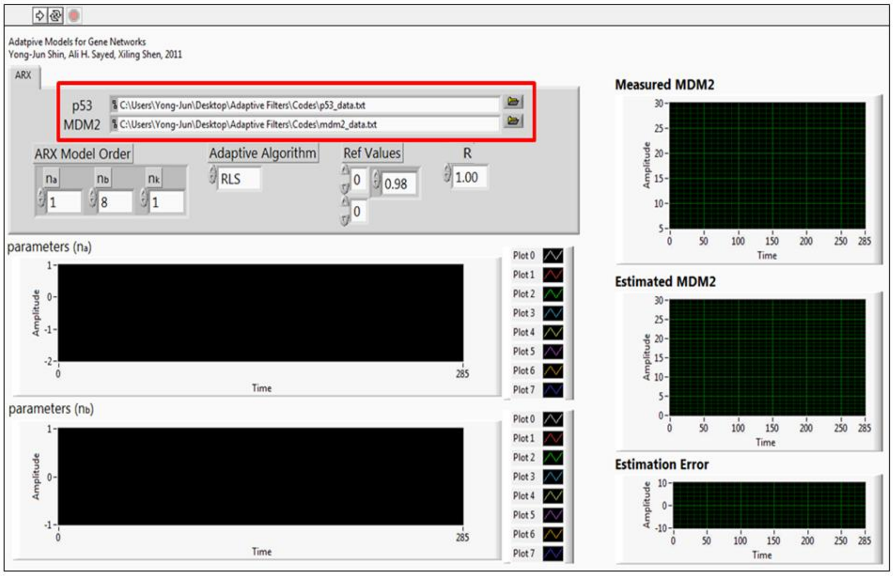

Figure S3. Load the data files.

To execute the application, it is required that LabVIEW 2010 or LabVIEW Run-Time Engine 2010 is installed on the computer. LabVIEW Run-Time Engine 2010 can be downloaded for free at http://joule.ni.com/nidu/cds/view/p/id/2087/lang/en. Three files needed for the simulated experiments are: AFGN.exe (**Supplementary Software S1**), p53_data.txt (**Supplementary Data S1**), and mdm2_data .txt (**Supplementary Data S2**).

1. Execute AFGN.exe and load the p53 and MDM2 data files (**Fig. S3**).

2. Select the ARX model order (n_a, n_b, and n_k). The default values are n_a = 1, n_b = 8, and n_k = 1.

3. Select an adaptive algorithm. There are four choices: LMS, NLMS, RLS, and KF (Kalman Filter).

4. Ref Values is a matrix and the value for each element can be inserted by selecting the row and column indices as shown in **Fig. S4**.

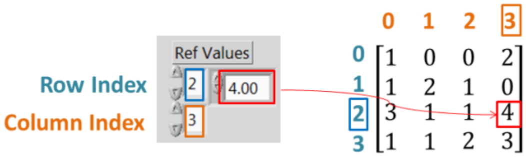

Figure S4. Inserting values into Ref Values.

a) LMS and NLMS: The first-row first-column (0, 0) value is the step-size μ.

b) RLS: The first-row first-column (0, 0) value is the forgetting factor λ.

c) KF: For KF, Ref Values becomes $\mathbf{Q}$, the *m* by *m* correlation matrix ($m = n_a + n_b$), which is related to the environmental uncertainty (biological noise) e_2. For our simulated experiments in the main text, we used

$$\mathbf{Q} = \sigma^2 \mathbf{I}$$

where σ is the standard deviation (chosen as 1) and $\mathbf{I}$ is the identity matrix (*m* by *m*).

5. The measurement noise variance *R* value is required only for KF. The default value is 1.

6. Run the application by clicking the arrow at the top (**Fig. S5**). Parameter tracking can be observed below the control panel.

7. In order to re-initialize the values to default, click [Edit] from the top menu and select [Reinitialize Values to Default].

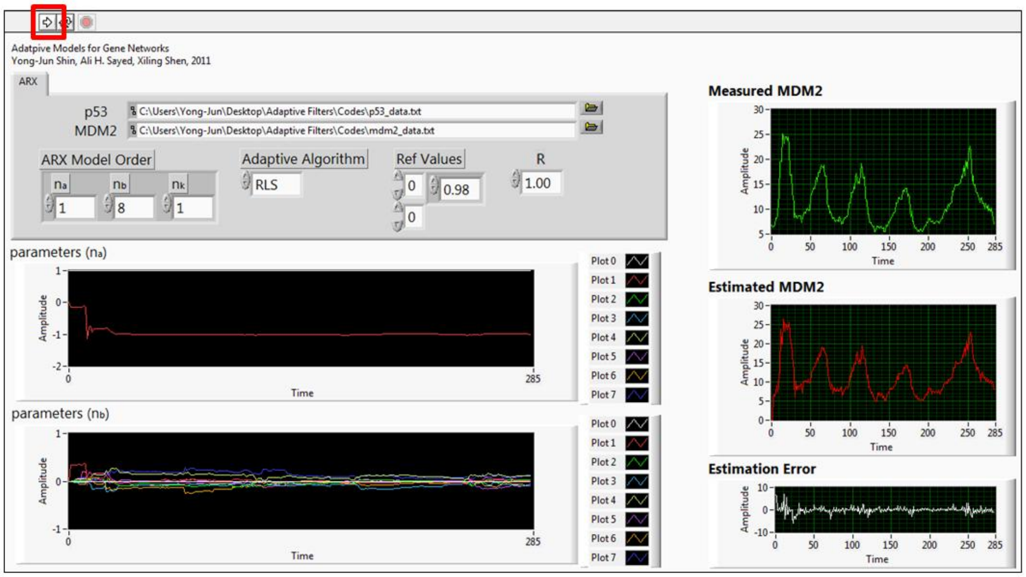

Figure S5. Running the application by clicking the arrow at the top.

Supplementary Note 5: Steps for reproducing Figure 2c and 2d

1. From [Edit] (top-level drop-down menu), select “Reinitialize Values to Default”.

2. Load data files as described in Supplementary Note 4.

3. Set the ARX Model Order as “n_a = **2**”, “n_b=**1**”, and “n_k = **2**”.

4. Choose "**NLMS**" for Adaptive Algorithm.

5. Change "Ref Values" from 0.98 to **0.1**.

6. Run the application by clicking the arrow at the top (**Fig. S5**).

References

1. Shin YJ, Bleris L. (2010) Linear control theory for gene network modeling. PLoS One 5(9): e12785.
2. Geva-Zatorsky N, Dekel E, Batchelor E, Lahav G, Alon U. (2010) Fourier analysis and systems identification of the p53 feedback loop. Proc Natl Acad Sci U S A 107(30): 13550-13555.
3. Ljung L, MathWorks I. (2007) System identification toolbox 7 : User's guide. Natick, Mass.: MathWorks, Inc.
